# Disruption of 5-hydroxytryptamine 1A receptor and orexin receptor 1 heterodimer formation affects novel G protein-dependent signaling pathways and has antidepressant effects in vivo

**DOI:** 10.1038/s41398-022-01886-1

**Published:** 2022-03-25

**Authors:** Rumin Zhang, Dandan Li, Huiling Mao, Xiaonan Wei, MingDong Xu, Shengnan Zhang, Yunlu Jiang, Chunmei Wang, Qing Xin, Xiaoyu Chen, Guorong Li, Bingyuan Ji, Maocai Yan, Xin Cai, Bo Dong, Harpal S. Randeva, Chuanxin Liu, Jing Chen

**Affiliations:** 1 Neurobiology Institute, Jining Medical University, Jining, China; 2 Department of Physiology, Shandong First Medical University, Taian, China; 3 School of Life Sciences, Shandong Normal University, Jinan, China; 4 School of Pharmacy, Jining Medical University, Shandong, China; 5 Department of Physiology, Weifang Medical University, Weifang, China; 6 Department of Cardiology, Shandong Provincial Hospital Affiliated to Shandong First Medical University, Jinan, China; 7 Division of Biomedical Sciences, Warwick Medical School, University of Warwick, Coventry, UK

**Keywords:** Molecular neuroscience, Neuroscience

## Abstract

G protein-coupled receptor (GPCR) heterodimers are new targets for the treatment of depression. Increasing evidence supports the importance of serotonergic and orexin-producing neurons in numerous physiological processes, possibly via a crucial interaction between 5-hydroxytryptamine 1A receptor (5-HT1AR) and orexin receptor 1 (OX1R). However, little is known about the function of 5-HT1AR/OX1R heterodimers. It is unclear how the transmembrane domains (TMs) of the dimer affect its function and whether its modulation mediates antidepressant-like effects. Here, we examined the mechanism of 5-HT1AR/OX1R dimerization and downstream G protein-dependent signaling. We found that 5-HT1AR and OX1R form constitutive heterodimers that induce novel G protein-dependent signaling, and that this heterodimerization does not affect recruitment of β-arrestins to the complex. In addition, we found that the structural interface of the active 5-HT1AR/OX1R dimer transforms from TM4/TM5 in the basal state to TM6 in the active conformation. We also used mutation analyses to identify key residues at the interface (5-HT1AR R151^4.40^, 5-HT1AR Y198^5.41^, and OX1R L230^5.54^). Injection of chronic unpredictable mild stress (CUMS) rats with TM4/TM5 peptides improved their depression-like emotional status and decreased the number of endogenous 5-HT1AR/OX1R heterodimers in the rat brain. These antidepressant effects may be mediated by upregulation of BDNF levels and enhanced phosphorylation and activation of CREB in the hippocampus and medial prefrontal cortex. This study provides evidence that 5-HT1AR/OX1R heterodimers are involved in the pathological process of depression. Peptides including TMs of the 5-HT1AR/OX1R heterodimer interface are candidates for the development of compounds with fast-acting antidepressant-like effects.

## Introduction

G protein-coupled receptor (GPCR) dimerization is crucial for various receptor functions, including agonist affinity, efficacy, trafficking, and specificity of signal transduction. GPCRs can form heterodimers and homodimers; the former is exemplified by orexin receptor 1 (OX1R) and kappa opioid receptor heterodimers, as well as by apelin receptor (APJ) and bradykinin 1 receptor heterodimers [1, 2]. In addition, APJ can also form homodimers [3]. GPCR dimers have specific functional characteristics that differ from those of the monomers. Indeed, the dimerization of GPCRs is an important way to regulate receptor function. For example, hetero-oligomerization of dopamine D2 receptors and somatostatin receptor type 5 increases their functional responses to the agonists dopamine and somatostatin [4]. In addition, heterodimerization of OX1R and cholecystokinin A receptor inhibits the migration of colon cancer cells in humans [5]. Many GPCR dimers are associated with pathological conditions such as schizophrenia, Parkinson’s disease, and drug addiction [6]. Consequently, GPCR homo and heterodimers are often preferentially targeted over the monomers when designing novel drugs [7–10].

5-Hydroxytryptamine (5-HT, serotonin) is an important signaling molecule that regulates and modulates several physiological and behavioral processes in the human body. In the central nervous system (CNS), 5-HT modulates processes associated with mood, perception, reward, anger, aggression, appetite, memory, sexual behavior, and attention [11]. Furthermore, 5-HT is involved in the pathogenesis of various psychiatric diseases, including depression, schizophrenia, and anxiety. There are 14 subtypes of the 5-HT receptor in humans, of which 13 are GPCRs, and five different genes encode the 5-HT1A, 1B, 1D, 1E, and 1F receptor subtypes. In general, the 5-HT1A receptor (5-HT1AR) couples with Gαi/o proteins and inhibits adenylate cyclase activity [12]. This receptor subtype has been well-studied, and its dysregulation is involved in disease states such as depression and anxiety [13].

In the CNS, orexins and orexin receptors regulate various behavioral and physiological responses, including arousal and narcolepsy, control of energy metabolism, food intake, drug reward, anxiety, and depression-related responses [14, 15]. Orexin-A and -B orchestrate their diverse effects by binding to and activating two GPCRs, orexin receptor 1 (OX1R) and OX2R, both of which are expressed widely in the CNS and can couple with Gαq, Gαs, and Gαi [16, 17]. Although orexin-A binds preferentially to OX1R, it can also bind to OX2R. Human OX1R forms both homodimers and heterodimers, the latter with, for example, growth hormone secretagogue receptor 1a or cannabinoid receptor type 1 [18, 19].

There are some overlaps between the functions and distributions of 5-HT1AR and OX1R in the CNS. For example, 5-HT inhibits orexin neurons directly by binding to 5-HT1AR and indirectly by facilitating GABAergic inhibitory inputs without affecting glutamatergic inputs [20]. In addition, expression of both 5-HT1AR and OX1R is associated with depression and anxiety. Recently, homodimerization has been reported for 5-HT1AR [21]. In addition, 5-HT1AR can interact with other GPCRs to form heterodimers, which may selectively modulate distinct intracellular signal transduction pathways [22–25]. However, the existence and distribution of the 5-HT1AR/OX1R heterodimer, as well as its structural interface and potential functions, remain controversial.

Here, we investigated whether 5-HT1AR and OX1R can heterodimerize in vitro and in vivo. Our findings suggest novel signal transduction upon heterodimerization of these receptors. Mass spectrometry and peptides, in which specific transmembrane domains (TMs) of 5-HT1AR and OX1R were fused with the human immunodeficiency virus trans-acting transcriptional activator (HIV TAT), were used to examine the formation of 5-HT1AR/OX1R heterodimers. We also examined the 5-HT1AR/OX1R dimer interface and its dynamics during receptor activation. Further mutational analyses confirmed the key residues important for the interaction interface. In in vivo experiments, we found that increased expression of the 5-HT1AR/OX1R heterodimer in the hippocampus and medial prefrontal cortex (mPFC) was related to depression and anxiety-like behaviors in rats. We then examined the effects of injecting specific 5-HT1AR and OX1R-TM peptides into the lateral ventricle of rats exposed to chronic unpredictable mild stress (CUMS). Our findings suggest that TM peptides provide an experimental basis for further research into the etiology of depression and represent new candidate small molecules that could be used to interfere with 5-HT1AR/OX1R dimerization for the treatment of depression.

## Materials and methods

Full details of the materials and methods are provided in the Supplementary Information.

### Animals

CUMS rat model procedures were performed as described previously [26]. To validate the efficacy of the CUMS protocol, weight gain was determined, and forced swim test (FST) and sucrose preference test (SPT) assessments were performed as described in previous reports [27, 28]. Rats in the CUMS plus TM peptide treatment group were injected with the corresponding TM peptide for 6 days (100 μg/8 μl). Rats in the CUMS group were injected with 8 μl normal saline on the same days, and the control group was left untreated. The study was approved by the local ethics board of Jining Medical University and met the standards of the Guide for the Care and Use of Laboratory Animals issued by the Ministry of Science and Technology of the People’s Republic of China in 2006.

### Co-immunoprecipitation

HEK293 cells were co-transfected with Myc-5-HT1AR and 3HA-OX1R or a vector control and lysed 48 h later. After centrifuging for 15 min at 4 °C and 16,000 × *g*, whole cell lysates were incubated with an anti-HA antibody and Protein G sepharose beads for 4 h with gentle rotation at 4 °C. The beads were then washed four times with cell lysis buffer. Precipitates were eluted with 4× SDS-PAGE sample buffer and analyzed by western blotting for anti-Myc immunoreactivity.

### Double-immunofluorescence staining

For 5-HT1AR and OX1R localization experiments, rat brains were fixed in 4% formaldehyde overnight, and then embedded in paraffin blocks and sectioned using a microtome. The hippocampal sections were incubated with goat anti-5-HT1AR (1:100; Abcam, Cambridge, UK) and rabbit anti-orexin receptor 1-ATTO-488 (1:60); Alomone Labs, Jerusalem, Israel) antibodies overnight at 4 °C. After washing with PBS, each section was incubated with a Cy3-conjugated goat anti-rabbit IgG antibody (1:100; Boster Biological Technology, Pleasanton, US) at 25 °C for 2 h. After a further wash with PBS, each section was incubated with DAPI (1:100,000; Invitrogen, California, US) at 25 °C for 10 min. The stained sections were examined under a Leica DMRE laser scanning confocal microscope (Leica, Milton Keynes, UK).

### In situ proximity ligation assay (PLA)

Interactions of 5-HT1AR and OX1R were detected in the native tissue using the Duolink II in situ PLA detection kit (Sigma-Aldrich, St. Louis, MO, USA), following the supplier’s instructions. Rat brain (hippocampus and mPFC) sections of 5 μm thickness were used in the PLA experiments. A mixture of the primary antibodies [goat polyclonal anti-5-HT1AR (1:100; Sigma-Aldrich Gillingham, UK) and rabbit polyclonal anti-OX1R (1:100; Abcam)] was used to detect 5-HT1AR/OX1R heterodimers, and PLA probes were used to detect the goat and rabbit antibodies. Punctate fluorescent signals were indicative of proximity (∼10 nm) of the 5-HT1AR and OX1R protomers. The formation of heterodimers was then confirmed by laser scanning confocal microscopy with an apochromatic 63× oil-immersion objective, and 405 and 561 nm laser lines. Similar methods were used to identify 5-HT1AR/OX1R heterodimers in HEK293 cells transfected with vectors expressing each protomer. HEK293 cells expressing OX1R alone were used as negative controls.

To examine the effects of TM peptides on 5-HT1AR/OX1R dimerization, HEK293 cells were transfected with the pcDNA3.1-5-HT1AR and pcDNA3.1-OX1R plasmids in 12-well plates. After 24 h, the cells were distributed into 6-well plates and treated for 6 h with HIV TAT-fused TM peptides (10 μM) corresponding to TM4 or TM5 of 5-HT1AR, or TM1 or TM5 of OX1R, and then assayed for dimerization as described above.

### Bioluminescence resonance energy transfer (BRET) saturation assay

To monitor constitutive 5-HT1AR/OX1R interactions, HEK293 cells were transfected with 5-HT1AR-Rluc and OX1R-EGFP or OX1R-Rluc and 5-HT1AR-EGFP plasmids at ratios of 1:1, 1:2, 1:3, 1:4, 1:5, and 1:6. Coelenterazine h was then added, and BRET measurements were taken using a Tristar LB941 plate reader with Rluc (400–475 nm) and EGFP (500–550 nm) filters.

### Fluorescence resonance energy transfer (FRET)

To obtain calibration coefficients and eliminate excitation and emission crosstalk, 5-HT1AR-ECFP and OX1R-EYFP were transfected into HEK293 cells as donor and acceptor channels, respectively. After 24 h, FRET signals were detected with a FRET kit using a Leica AM TIRF MC System. FRET efficiency (EA) was calculated as shown in the equation below; A, B, and C correspond to the intensities of the three signals (donor, FRET, and acceptor, respectively), and α, β, γ, and δ are the calibration factors generated by the acceptor-only and donor-only references:

$${{{\mathrm{EA}}}}(i) \,=\, \frac{{{{{\mathrm{B}}}} \,-\, {{{\mathrm{A}}}} \,\times\, \beta \,-\, {{{\mathrm{C}}}} \,\times\, (\gamma \,-\, \alpha \,\times\, \beta )}}{{{{{\mathrm{C}}}} \,\times\, (1 \,-\, \beta \,\times\, \delta )}}$$

### Design and synthesis of TM peptides

Wild-type and mutant human 5-HT1AR and OX1R-TM peptides were custom-synthesized; their primary sequences are shown in Table 1. Because HIV TAT is an excellent transporter for delivery purposes. peptides were fused to TAT mediates the transduction of peptides into target cells. HIV TAT was fused at the N-terminus of even-numbered TMs and at the C-terminus of odd-numbered TMs to ensure proper orientation of the inserted peptide.

**Table 1:** Amino acid sequences of synthetic peptides derived from the transmembrane domains of OX1R and 5-HT1AR.

**OX1R-TM**		**Molecular weight (Da)**
TM1	WVLIAAYVAVFLIALVGNTLV**YGRKKRRQRRR**	3787.61
TM4	**YGRKKRRQRRR**ARGSILGIWAVSLAIMVPQAAV	3765.54
TM5	IYHSCFFIVTYLAPLGLMAMAY**YGRKKRRQRRR**	4066.88
TM6	**YGRKKRRQRRR** MLMVVLLVFALCYLPISVLNVLK	4134.23
TM6-L309A	**YGRKKRRQRRR** MLMVVLLVFA*A*CYLPISVLNVLK	4092.15
**5-HT1AR-TM**		**Molecular weight (Da)**
TM3	LFIALDVLCCTSSILHLCAIAL**YGRKKRRQRRR**	3874.75
TM4	**YGRKKRRQRRR**RRAAALISLTWLIGFLISIPPMLGWRTP	4692.70
TM5	DHGYTIYSTFGAFYIPLLLMLVL**YGRKKRRQRRR**	4566.41
TM6	**YGRKKRRQRRR**VKTLGIIMGTFILCWLPFFIVAL	4138.16
TM4-R151A	**YGRKKRRQRRR***A*RAAALISLTWLIGFLISIPPMLGWRTP	4607.40
TM6-M351A	**YGRKKRRQRRR** VKTLGII*A*GTFILCWLPFFIVAL	4078.04
TM6-L356A	**YGRKKRRQRRR** VKTLGIIMGTFI*A*CWLPFFIVAL	4096.08

Italic letter indicates replacement of the original amino acid by alanine.

### Mass spectrometry

Mass spectrometry was performed to identify 5-HT1AR/OX1R dimer interfaces in samples treated with TM peptides. HEK293 cells were transfected with 5-HT1AR or OX1R, and 48 h later, were treated with or without the indicated HIV TAT-TM fused peptides (4 μM) for 60 min at 37 °C. The 5-HT1AR or OX1R complex was analyzed using a matrix-assisted laser desorption/ionization-time of flight (MALDI-TOF) mass spectrometer [29].

### Statistical analysis

All data are shown as means ± SEM. Data are presented and analyzed using GraphPad Prism 5.0 software. Sigmoidal curves were fitted to the dose–response data using nonlinear regression. Statistical analysis was performed using one-way analysis of variance followed by Tukey’s multiple comparison post-test. Differences between the means were considered statistically significant at *P* < 0.05.

## Results

### 5-HT1AR and OX1R form heterodimers in vitro and in vivo

**Fig. 1:**
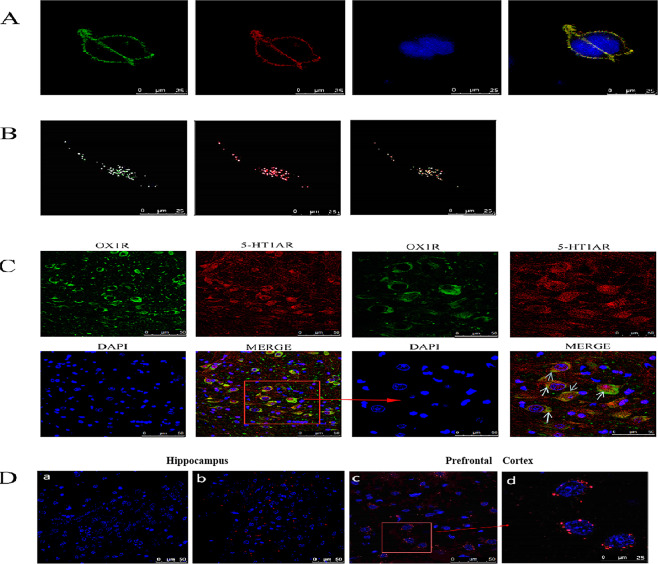
Co-expression and co-localization of 5-HT1AR and OX1R. The co-localization of 5-HT1AR/OXX1R in transfected HEK293 cells was analyzed by confocal microscopy (**A**). N-terminally Myc-tagged 5-HT1AR and C-terminally EGFP-tagged OX1R were expressed in HEK293 cells and visualized using laser confocal microscopy. The Cells images of 5-HT1AR (red) and OX1R (green) were merged to show regions of co-localization (yellow) on the cell membrane. Nuclei were stained with the DNA-specific dye DAPI (blue). Analysis of 5-HT1AR/OX1R -transfected HEK293 cells using total internal reflection fluorescence microscope (TIRFM) is shown in (**B**). In (**B**), the image on the left is the color of the 5-HT1AR display: green; the middle image is the color of the OX1R display: red; the right image is the color produced by the merge: yellow. The area where yellow exists is the area of receptor co-localization. The objective magnification is ×63. Double-immunofluorescence staining and confocal images to detect co-localization of 5-HT1AR/OX1R in the neuronal cells of the hippocampus are displayed in (**C**). Cells were processed for immunostaining using fluorescein (green)-conjugated rabbit anti-OX1R antibodies and rhodamine (red)-conjugated goat anti-5-HT1AR antibodies and analyzed by confocal microscopy. Images reveals the co-localization of 5-HT1AR/OX1R in yellow. **C** Shows OX1R immunoreactivity (green), 5-HT1AR immunoreactivity (red), DAPI (blue) and 5-HT1AR/OX1R co-localization (yellow). Scale bars represent 10 µm. Confirmation of 5-HT1AR/OX1R heterodimer formation in the mPFC and hippocampus of the rat using in situ PLA. Confocal microscopy images (**D**) are shown in which 5-HT1AR/OX1R heterodimers appear as red fluorescence spots. Cell nuclei were stained with DAPI (blue). The two pictures on the left of (**D**) are rat hippocampus, it shows nuclear and 5-HT1AR/OX1R heterodimers (red fluorescence spots), respectively. The two figures on the right of (**D**) are mPFC. The right image shows the partially enlarged cells in the left image. Scale bars represent 50 μm. Representative images from four independent experiments (*n* = 4) are shown as above.

Cell surface localization of 5-HT1AR and OX1R was unaffected by their co-expression in HEK293 cells. Immunofluorescence and total internal reflection fluorescence microscopy analyses of HEK293 cells co-expressing Myc-tagged 5-HT1AR and EGFP-tagged OX1R confirmed that both proteins were co-localized on the cell membrane (Fig. 1A, B). This figure showed that labeling is observed in neurons in the rat hippocampus. In vivo, Here, double-immunofluorescence staining, we found that the neurons in the rat hippocampus endogenously co-express 5-H1AR and OX1R, and there is co-localization between 5-H1AR and OX1R (Fig. 1C, Merge). On the right side of the cell image (Fig. 1C (Merge), there is a partially enlarged unit showing the clearly visible nucleus (blue). The cell imaging showed significant co-localization of the 5-H1AR and OX1R on the cell membrane (indicated by arrows). This study laid a solid foundation for the further study of 5-H1AR and Ox1RT dimerization. In addition, to confirm the in vivo findings, an in situ PLA showed that 5-HT1AR and OX1R form heterodimers in the rat mPFC and hippocampus tissue (Fig. 1D). The two pictures on the left of Fig. 1Da, b are rat hippocampus, Fig. 1Da shows cell nucleus in blue (DAPI). It shows nuclear and 5-HT1AR/OX1R heterodimers (red fluorescence spots, Fig Fig. 1Db. The two figures on the right of Fig. 1D are mPFC. Each image (Fig. 1Dc, d also shows 5-HT1AR/OX1R heterodimers (red fluorescence spots). The right image (Fig. 1Dd shows the partially enlarged cells in the left image. We showed the co-localization and co-expression of 5-H1AR and OX1R in rat hippocampus and mPFC. These results indicate that there are 5-H1AR and OX1R in the hippocampus-mPFC circuit organization, which may play an important role in the neurobiology of depression.

**Fig. 2:**
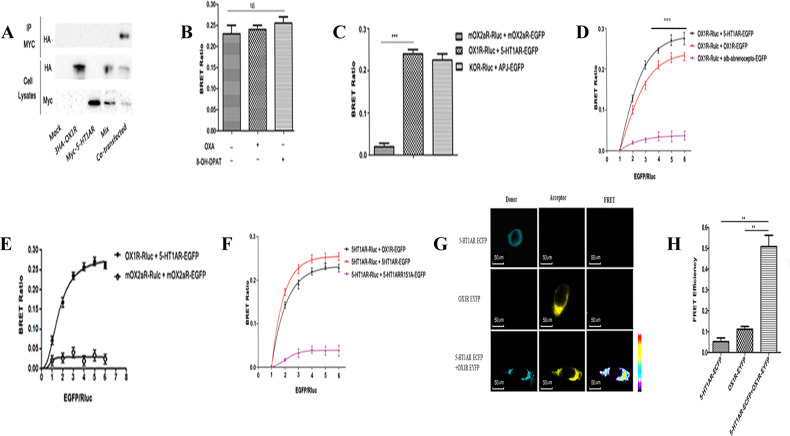
Heterodimerization of 5-HT1AR/OX1R examined with Co-IP, BRET and FRET. **A** HEK293 cells were either not transfected (mock) or transfected with Myc-5-HT1AR, 3HA-OX1R, or both (co-transfection). As negative control, samples containing either HEK293 Myc-5-HT1AR or HEK293-3HA-OX1R cells were mixed (Mix). Cell lysates were immunoprecipitated with anti-Myc agarose beads and immunoblotted with anti-HA antibody (upper panel). Cell lysates were examined by immunoblotting with either an anti-HA or anti-Myc antibody (lower panels) (**A**). The data represent means ± SEM of four independent experiments (*n* = 4). Heterodimerization of 5-HT1AR/OX1R was measured by BRET (**B**). Effects of 8-OH-DPAT or orexin-A on the BRET ratio. HEK293 cells were co-transfected with OX1R-Rluc and 5-HT1AR-EGFP plasmids (1:3). After 24 h of transfection, the Rluc substrate Coelenterazine h was added for 5 min, and the cells were treated with 8-OH-DPAT (100 nM) or orexin-A (100 nM) or vehicle for 10 min, BRET ratios were analyzed and are expressed as the mean ± SEM of four experiments (*n* = 4) (**B**). **C** BRET ratios were analyzed and are expressed as the means ± SEM of four experiments (*n* = 4). ***p < 0.001, OX1R-Rluc + 5-HT1AR-EGFP versus a negative control group (mOX2αR-Rluc + mOX2αR-EGFP), as a positive control group (κOR-Rlue + APJ-EGFP) (**C**). **D**, **E**, **F** BRET saturation assay HEK293 cells were co-transfected with a constant amount of the OX1R-Rluc (**D**, **E**) or 5-HT1AR-Rluc (**F**) construct, each at 0.15 μg/well, and increasing amounts of the EGFP construct (0.15–0.9 μg/well). Calculated BRET ratios were plotted relative to total fluorescence/luminescence ratios and data were analyzed by nonlinear regression curve fitting (one site–specific binding) using GraphPad Prism. BRET ratios were analyzed and expressed as means ± SEM of four experiments (*n* = 4). **G** FRET imaging of constitutive 5-HT1AR/OX1R heteromeric interactions in living cells. HEK293 cells were transiently transfected with plasmids encoding (a) 5-HT1AR-ECFP (donor), (b) OX1R-EYFP (acceptor), (c) 5-HT1AR-ECFP and OX1R-EYFP. Left panels: ECFP center panels, EYFP right panels:, corrected FRET. **H** 5-HT1AR/OX1RAPJ heterodimer FRET efficiency. Four independent experiments were performed with duplicate samples and the results were expressed as the mean ± SEM of four experiments (*n* = 4) (**p < 0.01 vs. other groups).

We investigated whether 5-HT1AR and OX1R can form heterodimers in vitro. A co-immunoprecipitation analysis of HA-tagged OX1R and Myc-5-HT1AR in co-transfected HEK293 cells revealed that immunoreactive bands were only detected by the anti-HA and anti-Myc antibodies when both receptors were co-expressed (Fig. 2A). Next, we examined the bioluminescence resonance energy transfer (BRET) ratios in HEK293 cells expressing OX1R-Rluc and 5-HT1AR-EGFP that were treated with the OX1R agonist orexin-A or the 5-HT1AR agonist 8-OH-DPAT (Fig. 2B). The BRET ratios of the treated cells were not significantly different from those of the unstimulated cells, indicating that the formation of 5-HT1AR/OX1R heterodimers was agonist-independent. By contrast, the negative controls, mOX2αR-Rluc and mOX2αR-EGFP [30] produced linear BRET ratios, indicating a non-specific interaction (Fig. 2C). Notably, OX1R homodimerization compared with HEK293 cells co-expressing 5-HT1AR and OX1R, Bret ratio was reduced slightly (Fig. 2D). In the negative control group, co-expression of the α1b-adrenoceptor-EGFP and OX1R-Rluc in cells resulted in a low BRET ratio (Fig. 2D), which is consistent with a previous report that BRET signals are low in cells co-expressing OX1R-Rluc and α1b-adrenoceptor-EYFP [31]. In addition, a BRET saturation assay of HEK293 cells that were co-transfected with 5-HT1AR-EGFP and OX1R-Rluc produced a saturation curve, confirming the interaction of 5-HT1AR and OX1R in vitro (Fig. 2E). BRET saturation assays also confirmed that constructs with labels in the “opposite” configuration (i.e., 5-HT1AR-Rluc and OX1R-EGFP) were able to dimerize when expressed in HEK293 cells (Fig. 2F).

Next, we transfected HEK293 cells with 5-HT1AR-ECFP alone, OX1R-EYFP alone, or 5-HT1AR-ECFP and OX1R-EYFP (Fig. 2G, interaction sites are shown in yellow). Low fluorescence resonance energy transfer (FRET) signals were observed in cells transfected with either construct alone. However, an enhanced FRET signal was seen in cells expressing 5-HT1AR-ECFP and OX1R-EYFP, providing further evidence that 5-HT1AR and OX1R can form dimers (Fig. 2G). Normalized FRET values were calculated as described in the Materials and methods section, and the FRET efficiency of the 5-HT1AR/OX1R heterodimer is shown in Fig. 2H.

### Heterodimerization of 5-HT1AR and OX1R affects downstream signaling pathways

#### Heterodimerization of 5-HT1AR and OX1R increases intracellular cAMP levels

**Fig. 3:**
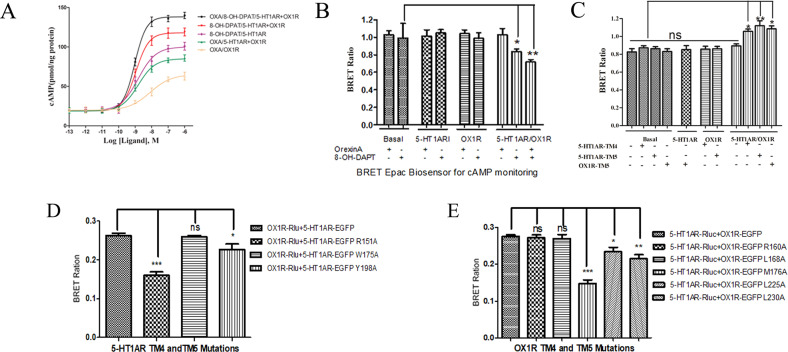
Evaluation of cellular cAMP levels by 5-HT1AR and OX1R heterodimer activation and detection of a series of 5-HT1AR-TM4, TM5 and OX1R-TM4, TM5 mutants by BRET. **A** ELISA assay for cAMP levels. HEK293-5-HT1AR, HEK293-OX1R, and HEK293-5-HT1AR/OX1R cells were stimulated with forskolin (10 μM) in the absence or presence of various concentrations of 5-HT1AR agonist 8-OH-DPAT (0.001–1000 nM) and/or OX1R agonist orexin-A (0.001–1000 nM). Intracellular cAMP concentrations were measured using an ELISA assay. Data are represented as means ± SEM (*n* = 3). The curve was fitted using nonlinear regression (log (agonist) vs. response—variable slope) in Prism 5.0. Data are represented as means ± SEM (*n* = 3). **B** BRET EPAC biosensor for cAMP monitoring. Measurement of BRET signals in HEK293 cells co-expressing the cAMP biosensor and 5-HT1AR, OX1R, or 5-HT1AR/OX1R and stimulated with 8-OH-DPAT only or 8-OH-DPAT and orexin-A. *p < 0.05; **p < 0.01. The data are expressed as the mean ± SEM (*n* = 4). **C** BRET EPAC biosensor and TM peptides for cAMP assay. HEK293 cells were co-transfected with the cAMP biosensor with 5-HT1AR, OX1R or 5-HT1AR/OX1R and incubated at 37 °C for 2 h with HIV TAT-fused TM peptides (10 μM) corresponding to TM5 of OX1R and TM4 or TM5 0f 5-HT-1AR stimulated with 8-OH-DPAT or orexin-A or 8-OH-DPAT and orexin-A. BRET ratios were analyzed and expressed as means ± SEM of four experiments (*n* = 4) (one-way analysis of variance: ns not significant, *p < 0.05; **p < 0.01 vs. basal group). **D**, **E** Experimental evaluation of 5-HT1AR-TM4 and TM5: R151A, W175A, Y198A (**D**), and OX1R-TM4 and TM5: R160A, L168A, M176A, L225A, L239A (**E**) mutants using BRET. Four independent experiments were performed with triplicate samples and the results were expressed as the mean ± SEM of four experiments (*n* = 4) (***p < 0.001, versus WT 5-HT1AR/OX1R dimer group).

The effects of 5-HT1AR/OX1R dimerization on intracellular cAMP concentrations were measured using an ELISA assay. Specifically, cAMP accumulation was compared in HEK293 cells expressing 5-HT1AR alone, OX1R alone, and 5-HT1AR/OX1R. The cells were stimulated with forskolin (10 μM) in the absence or presence of various concentrations of 8-OH-DPAT (0.001–1000 nM) and/or orexin-A (0.001–1000 nM). The cAMP level in cells expressing 5-HT1AR/OX1R and treated with both ligands was markedly higher than that in the same cells treated with either ligand individually (Fig. 3A). A cAMP BRET biosensor analysis confirmed that co-treatment of HEK293 cells expressing 5-HT1AR/OX1R with 8-OH-DPAT or 8-OH-DPAT and orexin-A can reduce BRET ratio and increased the intracellular cAMP level (Fig. 3B). We measured cAMP levels after stimulation with 8-OH-DPAT or 8-OH-DPAT and orexin-A, and the results were high with orexin-A in the 5-HT1AR/OX1R co-expression group.

Next, HEK293 cells were co-transfected with 5-HT1AR/OX1R and the BRET EPAC biosensor for cAMP monitoring, and the transfected cells were incubated at 37 °C for 2 h with HIV TAT-fused peptides (10 μM) corresponding to TM5 of OX1R or TM4 or TM5 of 5-HT1AR. Subsequently, the cells were stimulated with 8-OH-DPAT alone, orexin-A alone, or both agonists. The increased intracellular cAMP levels seen in cells expressing 5-HT1AR and OX1R were reduced following treatment with the 5-HT1AR TM4 or TM5 or OX1R-TM5 peptides respectively, suggesting disruption of the heterodimers (Fig. 3C). Notably, treatment with TM peptide did not eliminate cAMP in transfected cells, suggesting that 5-HT1AR and OX1R have specific functional effects on cAMP levels, even in the absence of dimerization. Overall, these results suggest that TM peptide affects the formation of heterodimerization of 5-HT1AR and OX1R in vitro, rather than changing the cAMP of 5-HT1AR and OX1R monomers (Fig. 3C).

#### Heterodimerization of 5-HT1AR and OX1R increases the activities of CRE and SRF-RE

Reporter gene assays offer a simple solution for the study and characterization of receptor/G protein coupling. The activities of cAMP response element (CRE), nuclear factor of activated T-cells response element (NFAT-RE), serum response element (SRE), and serum response factor response element (SRF) were assessed in HEK293 cells expressing 5-HT1AR alone, OX1R alone, or 5-HT1AR/OX1R (Supplementary Fig. 1A–D). Following stimulation with 8-OH-DPAT alone or 8-OH-DPAT and orexin-A, cells expressing 5-HT1AR/OX1R heterodimers displayed enhanced specific response elements (CRE and SRF, Supplementary Fig. 1A, B) causing Gαs- and Gα12/Gα13- G-dependent downstream signal transduction pathways. The downstream signaling activities of most GPCRs are terminated by phosphorylation and subsequent binding of β-arrestin proteins; thus, we examined whether heterodimerization of 5-HT1AR and OX1R would affect the recruitment of β-arrestins. Stimulation of HEK293 cells expressing 5-HT1AR-Rluc, untagged OX1R, and β-arrestin1-EGFP or β-arrestin2-EGFP with 100 nM 8-OH-DPAT or 100 nM orexin-A resulted in a robust and continuous increase in the ligand-induced BRET signal (Supplementary Fig. 1E, F), indicating that 5-HT1AR/OX1R heterodimerization does not alter the recruitment of β-arrestins to the complex.

### TM4/TM5 and TM6/TM6 form the interface for 5-HT1AR/OX1R dimerization

#### Mass spectrometry analyses

TMs are crucial for creating head-to-head interfaces in class A GPCR dimers [32, 33]. The sequences and molecular weights of the seven TMs in 5-HT1AR and OX1R are shown in Table 1. To identify dimerization interfaces in the TMs of 5-HT1AR and OX1R, the effects of cell-penetrating interference peptides containing the sequences of the hydrophobic transmembrane helices on 5-HT1AR/OX1R heterodimer formation were examined by MALDI-TOF mass spectrometry.

These analyses revealed that 5-HT1AR + OX1R-TM4, 5-HT1AR + OX1R-TM6, and OX1R + 5-HT1AR-TM6 dimers were not (Supplementary Figs. 2, 1A, C, F), Whereas 5-HT1AR + OX1R-TM5, OX1R + 5-HT1AR-TM4, and OX1R + 5-HT1AR-TM5 dimers were formed (Supplementary Figs. 2, 1B, D, E) in the inactive state (absence of ligand). These findings indicate that the structural interface of the 5-HT1AR/OX1R dimer consists of a combination of TM4 and TM5 in 5-HT1AR and TM5 in OX1R, which are likely bound within themselves in the inactive state.

We then examined the dynamics of this structural interface following stimulation of the heterodimer with 8-OH-DPAT or orexin-A. In this case, the OX1R + 5-HT1AR-TM6 and 5-HT1AR + OX1R-TM6 dimers were detected by mass spectrometry (Supplementary Fig. 2B, C, F), suggesting that the structural interface of the 5-HT1AR/OX1R heterodimer changes from TM4/TM5 to TM6 in the active conformation.

Next, mass spectrometry analyses of 5-HT1AR/OX1R heterodimers containing various point mutations in the TMs were performed to identify the specific residues involved in dimer formation. The R151A mutation in 5-HT1AR-TM4 and the M351A mutation in 5-HT1AR-TM6 abolished the formation of a heterodimer with wild-type OX1R. By contrast, the L356A mutation in 5-HT1AR-TM6 did not affect the formation of a heterodimer with wild-type OX1R after agonist stimulation (Supplementary Figs. 2C, 3E). In addition, the L309A mutation in OX1R-TM6 abolished the interaction with wild-type 5-HT1AR (Supplementary Figs. 2, 3F).

#### BRET assays

The BRET ratios of HEK293 cells expressing 5-HT1AR and OX1R were reduced significantly after incubation with HIV TAT-fused 5-HT1AR-TM4, 5-HT1AR-TM5, and OX1R-TM5 peptides, but were not affected by incubation with the OX1R-TM1 peptide. These findings suggest that the 5-HT1AR-TM4, 5-HT1AR-TM5, and OX1R-TM5 peptides impaired the heterodimerization of 5-HT1AR and OX1R in vitro (Supplementary Fig. 3A, B). In addition, in agonist-stimulated cells, the BRET ratios were reduced significantly by incubating with the HIV TAT-fused 5-HT1AR-TM6 peptide (Supplementary Fig. 3C).

The importance of TM4, TM5, and TM6 to formation of the 5-HT1AR/OX1R heterodimer interface was confirmed by point mutation of specific residues that have been reported previously to mediate receptor dimerization [34]. Briefly, we generated 31 mutant receptors that harbored mutations of various outward-facing and hydrophobic residues (Table 2). Proper membrane localization is a prerequisite for BRET, so the possibility of a decreased BRET ratio caused by incorrect 5-HT1AR/OX1R localization was excluded by observing the immunofluorescence of all mutants prior to BRET measurements. Notably, the 5-HT1AR R151A^4.40^ and OX1R M176A^4.57^ TM4 mutants and the 5-HT1AR Y198A^5.41^ and OX1R L230A^5.54^ TM5 mutants exhibited markedly lower BRET signals than the wild-type proteins, highlighting the significance of TM4 and TM5 to the formation of the dimer interface in the basal or inactive state (Fig. 3D, E). In particular, we found that the 5-HT1AR R151A^4.40^ and OX1R M176A^4.57^ mutations had the greatest disruptive effect on the interaction and heterodimerization of 5-HT1AR and OX1R. Following agonist stimulation, the BRET signals of the dimers containing the 5-HT1AR M351A^6.41^ and OX1R L309A^6.49^ TM6 mutants were 50% lower than those of the wild-type dimer (Supplementary Fig. 3D, E). Notably, agonist stimulation of the dimer harboring the 5-HT1AR L356A^6.46^ TM6 mutant increased the BRET signal (Supplementary Fig. 3D).

**Table 2:** Constructing point mutations for exploring the residues mediating 5-HT1AR and OX1R interaction.

5-HT1AR mutants	OX1R mutants
TM4 R151A^4.40^, 40 R152^4.41^, W175A^4.64^	TM4 R159A^4.40^, R160A^4.41^, I165A^4.46^, L166A^4.47^, I168A^4.49^, W169A^4.50^, L173A^4.54^, M176A^4.57^
TM5 Y198A^5.41^, Y215F^5.58^, Y224F^5.60^	TM5 Y215F^5.38^, Y224F^5.47^, L225A^5.48^, L228A^5.51^, L230A^5.54^
TM6 L347A^6.37^, I349A^6.39^, I350A^6.40^, M351A^6.41^, W355A ^6.45^, L356A^6.46^, L359A^6.49^	TM6 L300A^6.37^, V304A^6.41^, L309A^6.54^, L317A^6.54^, L320A^6.57^

#### Proximity ligation assay (PLA)

**Fig. 4:**
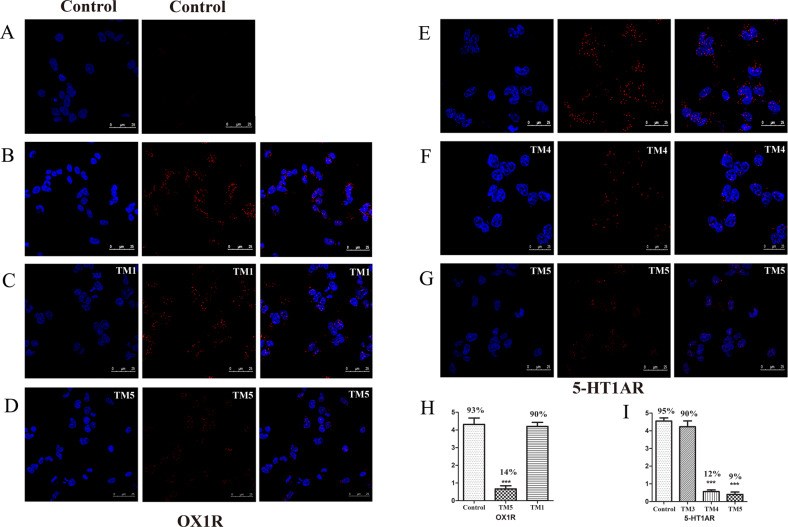
The role of TM peptides and in situ proximity ligation (PLA) confirmed 5-HT1AR/OX1R heterodimers. Detection of 5-HT1AR/OX1R heterodimers in HEK293 cells. Confocal microscopy images from PLA experiments performed in HEK293 cells expressing 5-HT1AR alone (**A**) or both 5-HT1AR/OX1R. HEK293 cells expressing 5-HT1AR/OX1R were treated with vehicle (**B**), HIV TAT-fused TM peptides (10 μM) corresponding to TM1 (**C**) or TM5 (**D**) of OX1R. The co-transfected HEK293 cells were treated with 10 μM of TM3 (control, **E**) or TM4 (**F**) or TM5 (**G**) of 5-HT1AR. Heteromeric complexes appear as red fluorescence spots and cell nuclei in blue (DAPI). Scale bars: 25 μm. Quantification from PLA experiments: (**H**, **I**) values (in means ± SEM, *n* = 4) is expressed as the ratio between the number of red fluorescence spots and the number of cells showing spots, (50–100 cells from four independent preparations); % values represent the percentage of cells showing one or more red fluorescence spots; ***p < 0.001, as compared to control.

When the cells expressing 5-HT1AR alone were used as the negative control group in the PLA experiment, no red fluorescent spots were found in the measurement (Fig. 4A). Red fluorescence spots were detected in HEK293 cells co-expressing 5-HT1AR and OX1R, indicating successful formation of the heterodimer (Fig. 4B). Pre-incubation of HEK293 cells co-expressing 5-HT1AR/OX1R with an OX1R-TM1 peptide (Fig. 4C) or OX1R-TM5 peptide (Fig. 4D), the latter significantly reduces the amount of PLA product. However, pre-incubation with a 5-HT1AR-TM3 peptide (Fig. 4E) did not change the number of PLA products. The pre-incubation with a 5-HT1AR-TM4 or -TM5 peptide (Fig. 4F, G) reduced the number of PLA products markedly Quantification of the PLA data is shown in Fig. 4H, I.

### Injection of 5-HT1AR/OX1R-TM peptides increases the amounts of proteins associated with antidepressant effects in the rat mPFC and hippocampus

**Fig. 5:**
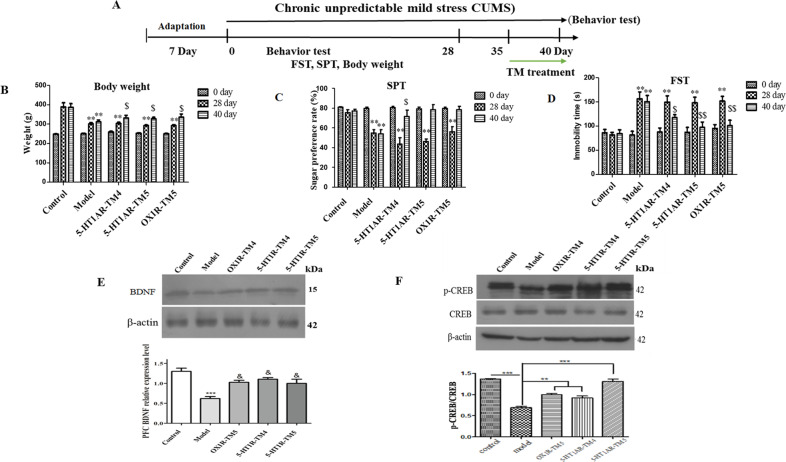
CUMS-induced depressive-like behavior could be improved by TM peptide treatment. Six days after injection of TM into the lateral ventricles of the CUMS model rats, the behavioral changes were compared with those of the control group and the CUMS model rats without injection of TM. **A** Schematic diagram of the experimental design for the CUMS paradigm. **B** Body weight. **C** Effects of TM treatment on the SPT in CUMS-exposed rat. **D** Effects of TM treatment on the immobility time in CUMS-exposed rat in the FST. The results showed that compared with the CUMS model rats, the behavior of the CUMS model rats injected with TM was improved (asterisk symbol (*) indicates that compared with the control group, $ the CUMS model rats injected with TM compared with CUMS model group, **P < 0.01; $ < 0.05; $$ < 0.01). In rats, BDNF (**E**) and pCREB/CREB (**F**) were upregulated in the mPFC *N* = 10 per group; * in comparison the control group with the depression (model) group, and & the CUMS model rats injected with TM compared with the depression model (CUMS) group, **p < 0.01; ^&^*p* < 0.05. pCREB/CREB was upregulated in the mPFC. *N* = 10 per group; in comparison the control group with model (CUMS), TM peptide treatment compared with depression model (CUMS) group, *P < 0.05; **P < 0.01. The data represent mean ± SEM of three independent experiments.

Next, we investigated the role of the 5-HT1AR/OX1R heterodimer in the onset of depression in vivo. To this end, we used the CUMS method to model depression in rats and injected the rats with 5-HT1AR/OX1R-TM peptides to disrupt formation of the heterodimer (Fig. 5A).

The experimental groups included an untreated control group (*n* = 10), a CUMS control group in which rats were injected with saline (*n* = 10), and a CUMS treatment group in which rats were injected with 5-HT1AR/OX1R-TM peptides were divided into three groups, each group (*N* = 10). After 28 days of the CUMS protocol, we measured the weight gain of the rats and performed SPT and FST to determine whether the model was successful. Ten rats that were successfully modeled were selected for subsequent experiments. Subsequently, the rats received a single injection of saline or TM peptides into the lateral ventricle, and weight gain, SPT, and FST assessments were performed 6 days later.

Weight gain of the CUMS group was significantly lower than that of the control group (**P < 0.01); however, treatment of the CUMS model rats with TM peptides increased weight gain significantly (^$^*P* < 0.05) (Fig. 5B). Similarly, the sucrose preference of the CUMS group was significantly lower than that of the control group (**P < 0.01) but was increased significantly following TM peptide injection (^$^*P* < 0.05; ^$$^*P* < 0.01) (Fig. 5C). In addition, injection of the TM peptides reversed the CUMS-induced increase in the immobility time in the FST (Fig. 5D). Taken together, these results suggest that the 5-HT1AR/OX1R dimer may be involved in the occurrence and development of depression. Six days of treatment with TM peptides increased proteins associated with antidepressant onset. These antidepressant effects may be mediated by upregulation of BDNF levels (Fig. 5E) and enhanced phosphorylation and activation of CREB (Fig. 5F) in the mPFC.

**Fig. 6:**
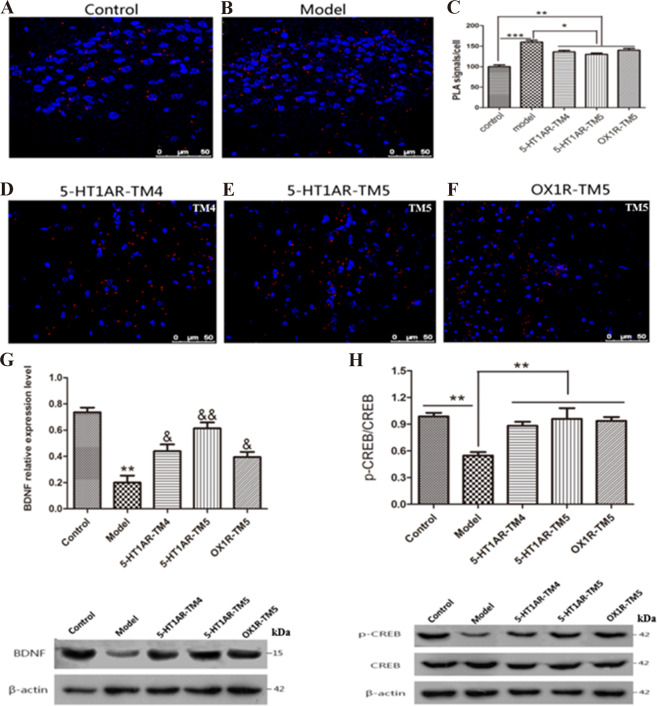
TM peptide treatment in rat hippocampus interferes with 5-HT1AR/OX1R heterodimer formation and added proteins associated with antidepressant-like effects. Image of the PLA signal (red fluorescence spots) for a 5-HT1AR/OX1R heterodimer in rat hippocampus tissue, Negative control group (**A**), CUMS model group (**B**). CUMS model rats were treated with 5-HT1AR-TM4 (**D**), 5-HT1AR-TM5 (**E**), and OX1R-TM5 (**F**) for 6 days or with saline in the control group. Nuclei visualized with DAPI staining (blue). In situ PLA was performed on sections and the number of PLA signals per image was quantified (**C**). The results were normalized to the mean value of the control group. The data represent mean ± SEM of four independent experiments (*n* = 4). Statistical analysis was performed by one-way ANOVA *p < 0.05; **p < 0.01; ***p < 0.001. Scale bar represents 50 μm. CUMS model rats were treated with 5-HT1AR-TM4, 5-HT1AR-TM5, and OX1R-TM5 (100 μg/8 μl) for 6 days. In the rat hippocampus, increased proteins associated with antidepressant-like effects was seen in Fig. 6. In rats, BDNF was upregulated in the hippocampus (**G**). *N* = 10 per group; * in comparison the control group with the depression (model) group, and & the TM peptides compared with the depression model (CUMS) group, **p < 0.01; ^&^*p* < 0.05; ^&&^*p* < 0.01. pCREB/CREB was upregulated in the hippocampus (**H**). *N* = 10 per group; in comparison the control group with model (CUMS), TM peptide treatment compared with depression model (CUMS) group, *P < 0.05; **P < 0.01. The data represent mean ± SEM of four independent experiments (*n* = 4).

To examine the role of the 5-HT1AR/OX1R heterodimer in the onset of depression further, we used a PLA to assess the amount of dimer formed in the rat hippocampus. The PLA signal representing the 5-HT1AR/OX1R dimer in the hippocampus was markedly higher in the CUMS group than in the control group (Fig. 6A, B). Next, in situ PLA signals were quantified as the number of blobs per cell in CUMS rats injected with the 5-HT1AR-TM4, 5-HT1AR-TM5, or OX1R-TM5 peptide compared with that in the control and CUMS groups (Fig. 6C). Compared with CUMS rats injected with 5-HT1AR-TM4 (Fig. 6D), 5-HT1AR-TM5 (Fig. 6E), or OX1R-TM5 (Fig. 6F), the PLA signal number of 5-HT1AR/OX1R heterodimer was lower.

Increased amounts of BDNF protein associated with antidepressant-like effects were seen in the hippocampus after rats were treated with 5-HT1AR-TM4 (2.66 mM), 5-HT1AR-TM5 (2.74 mM), or OX1R-TM5 (3.07 mM) respectively for 6 days (Fig. 6G). In addition, the level of BDNF and the phosphorylation and activation of CREB were upregulated after treatment with the TM peptides (Fig. 6H).

## Discussion

Although the formation of a 5-HT1AR/OX2R heterodimer has been reported previously, no study has revealed the characteristics of human 5-HT1AR/OX1R heterodimers. Here, we have demonstrated the formation of constitutive and functional 5-HT1AR/OX1R heterodimers in vitro and in vivo. Our results demonstrate that 5-HT1AR/OX1R heterodimers are formed in recombinant cell systems and neuronal cells of the rat mPFC (Supplementary Fig. 4) and hippocampus.

GPCR dimerization or multimerization is physiologically and functionally relevant, and often acts as the first step in the induction of intracellular signals following ligand binding [35, 36]. For example, heteromultimerization of cannabinoid receptor type 1 and OX1R generates a unique complex in which both protomers are regulated by orexin-A [19]. 5-HT1AR and 5-HT7R can form heterodimers in vitro and in vivo, and this heterodimerization reduces Gαi protein coupling of 5-HT1AR without affecting coupling of 5-HT7R to the Gαs protein [22]. 5-HT1AR and OX1R can also interact with other GPCRs to form heterodimers; thus, heterodimerization provides an additional mechanism to regulate cellular processes through the fine tuning of receptor-mediated signaling. Although 5-HT1AR and OX1R reportedly couple with Gαi and Gαq, respectively, we found that stimulation of 5-HT1AR/OX1R in HEK293 cells increased the coupling of the Gαs and Gα12/Gα13 subunits, indicating that these G protein subunits may be activated by binding of ligands to the 5-HT1AR/OX1R heterodimer. In line with this finding, the intracellular cAMP level was increased by double ligand stimulation of HEK293 cells expressing the 5-HT1AR/OX1R heterodimer. Conversely, incubation of transfected HEK293 cells with 5-HT1AR-TM4, 5-HT1AR-TM5, or OX1R-TM5 reduced the ligand-induced increase in the cAMP level, which is consistent with our observation that 5-HT1AR-TM4, 5-HT1AR-TM5, and OX1R-TM5 impaired formation of the 5-HT1AR/OX1R heterodimer. Overall, these results suggest that binding of a ligand to the 5-HT1AR/OX1R heterodimer increases the activation of Gαs and Gα12/Gα13. 5-HT1AR/OX1R is recruited into these heteropolymer complexes in novel G protein-dependent signaling pathways.

We used TM peptide and MALDI-TOF mass spectrometry as methods to examine the function of regulating 5-HT1AR/ox1r heterodimer. The results revealed that peptides encoding 5-HT1AR-TM4 and 5-HT1AR-TM5 could bind to OX1R, and a peptide encoding OX1R-TM5 could bind to 5-HT1AR, suggesting that these specific domains are involved in interface formation. GPCR dimers are a dynamic species with multiple forms and a dimerization interface that shifts during receptor activation and inactivation [37]. Here, we found that the structural interface of the active 5-HT1AR/OX1R dimer transforms from TM4 (5-HT1AR only) and TM5 (both protomers) in the basic or inactive state to mainly TM6 in the active conformation (Supplementary Fig. 5). Understanding that the 5-HT1AR/OX1R heterodimers are activated in this manner will aid the identification of new drug targets for small molecule interference in different dimer states.

In our previous study, we demonstrated that TM1, 2, 3, and 4 of APJ form the homodimer interface [3]. We propose that 5-HT1AR/OX1R protomers form type II dimers involving TM4 (5-HT1AR only), 5, and 6, and that TM6 plays a role in modulating 5-HT1AR/OX1R function. Although OX1R-TM4 did not bind to 5-HT1AR in our experiments (four replicates), we cannot rule out the possibility that this domain is involved in dimer formation, or that 5-HT1AR/OX1R may form asymmetric heterodimers. Similar to our findings presented here, the constitutive metabotropic glutamate receptor 2 homodimer undergoes a 45° rotation of each domain upon ligand binding, leading to a major change of the dimer interface from TM4 and TM5 in the inactive state to TM6-TM6 in the active conformation, revealing a key step in class C GPCR activation [38]. In addition, the structure of the chemokine receptor CXCR4 homodimer is based on a TM4/5 interface localized to the upper regions of the helices [39].

Our findings suggest that TM peptides could have therapeutic potential, as they can disrupt dimerization and influence receptor function. Bulenger et al. [35] showed that a peptide derived from TM6 of the β2-adrenergic receptor (β2-AR) can disrupt the dimer and decrease receptor function [40] and we have shown previously that disruption of TM1, 2, 3, and 4 in APJ impairs homodimer formation [3]. Due to the high sensitivity and accuracy of mass spectrometry, this method provides useful information for understanding GPCR structure and conformational changes during dimer formation. Point mutation experiments revealed that the 5-HT1AR R151A^4.40^ and OX1R M176A^4.57^ TM4 mutants (Supplementary Fig. 3) and the 5-HT1AR Y198A^5.41^ and OX1R L230A^5.54^ TM5 mutants exhibited a reduced ability to dimerize, further supporting the involvement of TM4 and TM5 in the dimer 5-HT1AR/OX1R interface. In addition, the 5-HT1AR M351A^6.41^ and OX1R L309A^6.49^ TM6 mutants inhibited agonist-stimulated 5-HT1AR/OX1R dimer formation, suggesting that these residues are involved in dimer formation in the active state. Interestingly, the 5-HT1AR L356A^6.46^ TM6 mutation increased 5-HT1AR/OX1R dimerization following agonist stimulation. In addition, a mass spectrometry analysis revealed that the 5-HT1AR L356A mutant peptide was able to bind to OX1R following agonist stimulation. To our knowledge, this is the first report that the 5-HT1AR-L356A^6.46^ mutation promotes 5-HT1AR/OX1R dimer formation. Further in vivo studies are required to clarify the specific role of this point mutation in increasing dimer formation.

The CUMS model used here is one of the most widely recognized animal models of depression [41]. Several behavioral effects that parallel symptoms of depression have been described in rats exposed to CUMS. Here, the FST and SPT were chosen as reliable methods for examining CUMS-induced behavioral effects. Using these parameters, as well as weight gain measurements, we examined the antidepressant-like effects of TM peptides in vivo. Six days after injection of one of three TM peptides (5-HT1AR-TM4, 5-HT1AR-TM5, and OX1R-TM5) into the lateral ventricle of CUMS rats, the CUMS-induced deficits in the SPT and increased immobility time in the FST were reversed. These TM peptides were designed to destroy the interface of the 5-HT1AR/OX1R heterodimer, suggesting that they inhibited the specific depression-related function of the GPCR dimer.

The hippocampus-PFC circuit plays a major role in stress and the neurobiology of depression. Accumulating evidence suggests dysregulation of synaptic plasticity in the etiology of depression, leading to synaptic weakening and neuronal atrophy in vulnerable brain regions such as the hippocampus and mPFC [42]. In addition, changes in the levels of neuropeptides and neurotrophic factors also play an important role in depression. Increased BDNF levels in the hippocampus and mPFC are associated with an antidepressant-like response in behavioral models of depression, providing a biomarker that corroborates the onset of these behavioral effects [43]. CREB impinges on the regulation of BDNF signaling, which exerts a highly circuit-specific influence on mood-related behaviors [44]. A leading hypothesis for the pathogenesis of depression is that CREB and BDNF play an important role in adaptation of the hippocampus to chronic stress and antidepressants [45]. Here, we found that treatment of CUMS rats with the 5-HT1AR-TM4, 5-HT1AR-TM5, and OX1R-TM5 peptides upregulated the BDNF level as well as phosphorylation and activation of CREB in the mPFC and hippocampus, in line with the observed alleviation of the CUMS-induced behavioral effects by these peptides. Notably, BDNF and pCREB/CREB were upregulated in the hippocampus and mPFC after just 6 days of TM peptide treatment; this rapid increase in the levels of these proteins is consistent with the effects of other putative fast-onset antidepressant treatments, such as short-term treatment with 5-HT2C receptor antagonists [46] and 5-HT4 receptor agonists [47]. Further research is needed to determine the correlation between the upregulation of BDNF and pCREB/CREB levels and the rapid initiation of antidepressant-like activities of the TM peptides used here.

Using the PLA method, we demonstrated that the number of endogenous 5-HT1AR/OX1R heterodimers in the rat hippocampus was markedly higher in the depression model group than in the control group. Alongside the improvement in depression-related effects, 6 days of treatment with the three TM peptides (5-HT1AR-TM4, 5-HT1AR-TM5, and OX1R-TM5) reduced the amount of 5-HT1AR/OX1R heterodimers in the hippocampus. Taken together, these findings provide direct evidence that 5-HT1AR/OX1R heterodimers are involved in the pathological process of depression. We suggest that TM peptides targeting the 5-HT1AR/OX1R heterodimer interface could be lead compounds for the development of fast-acting antidepressants.

In conclusion, this study advances current knowledge of the structure and function of 5-HT1AR/OX1R heterodimers. We have demonstrated that 5-HT1AR/OX1R can induce novel G protein-dependent signaling, and that heterodimerization of the protomers does not affect the recruitment of β-arrestins to the complex. We have also revealed the dynamics of the TM interface of the 5-HT1AR/OX1R heterodimer during receptor activation in vitro and in vivo. The antidepressant effects of TM peptides targeting the 5-HT1AR/OX1R dimer might be mediated by upregulation of BDNF levels in the mPFC and hippocampus, as well as by enhancement of CREB phosphorylation and activation. Our findings could aid the development of novel therapeutic drugs and small molecules that interfere with the interface of the 5-HT1AR/OX1R dimer in different states.

## Supplementary information

Supplementary Materials and Method
