# Supplementary material for: Disruption of 5-hydroxytryptamine 1A receptor and orexin receptor 1 heterodimer formation affects novel G protein-dependent signaling pathways and has antidepressant effects in vivo
Source: Transl Psychiatry. 2022 Mar 25;12:122. doi: 10.1038/s41398-022-01886-1 (PMC8956632; doi:10.1038/s41398-022-01886-1)
Supplement: Supplementary file 1 — Supplementary Materials and Method [file 41398_2022_1886_MOESM1_ESM.doc]

**Supplementary information for**

**Disruption of 5-hydroxytryptamine 1A receptor and orexin receptor 1 heterodimer formation affects novel G protein-dependent signaling pathways and has antidepressant effects *in vivo***

Rumin Zhang^1#^, Dandan Li^1#, 4^, Huiling Mao^1#^, Xiaonan Wei^1#^，MingDong Xu^1#^, Shengnan Zhang^1^, Yunlu Jiang^1^, Chunmei Wang^1^, Qing Xin^1^, Xiaoyu Chen^2^，Guorong Li^3^, Bingyuan Ji^1^, Maocai Yan^5^, Xin Cai^6^, Bo Dong^7^, Harpal S. Randeva^8^, Chuanxin Liu^1^, Jing Chen^1, 8^*

1 Neurobiology Institute, Jining Medical University, Jining, China

2 Department of Physiology, Shandong First Medical University, Taian, China

3 School of Life Sciences, Shandong Normal University, Jinan, China.

4 Ningyang First people's Hospital, Shandong, China.

5 School of Pharmacy, Jining Medical University, Shandong China.

6 Department of Physiology, Weifang Medical University, Weifang, China.

7 Department of Cardiology Shandong Provincial Hospital Affiliated to Shandong First Medical University, Jinan, China.

8 Division of Biomedical Sciences, Warwick Medical School, University of Warwick, Coventry, United Kingdom

# Contributed equally to this work.

*Correspondence to: [jing.chen@warwick.ac.uk](mailto:jing.chen@warwick.ac.uk)

**Supplementary Materials and Methods**

**Animals**

Sprague Dawley rats, Male, weighing 180–200 g, 6-7 weeks old were purchased from Beijing Vital River Laboratories Co. (SCXK (Jing) 2011-0012). All animals were housed in groups of 3–5 per cage for at least 7 days before testing with food and water available ad libitum. Animals were maintained under standard laboratory conditions (12-hour light/dark cycle, 24 ± 1°C, and 45 ± 15% humidity). All animal protocols were approved by Jining Medical University and met the standards of the Guide for the Care and Use of Laboratory Animals issued by the Ministry of Science and Technology of the People's Republic of China in 2006. For each treatment group in this series of experiments, twelve rats were used. The chronic unpredictable mild stress (CUMS) procedures were performed as previously described. The animals were tested for weight and behavior (forced swim test (FST) and sucrose preference test (SPT)). The FST and SPT were performed after CUMS, as described in the previous reports, to validate the CUMS procedure.

**Method of administration**

In order to avoid the failure of modelling rats to affect the results of this experiment, we performed behavioural tests at 0, 28 and 40 days of stimulation, and each group. Rats with similar behaviours and successful modelling for TM treatment. Rats were randomly assigned into 5 groups, with 10 rats per group: (1) Control group: without treated. (2) CUMS group: rat was injected with 8μl normal saline (3) CUMS + 5-HT1AR-TM4 treat group (4) CUMS + 5-HT1AR-TM5 treat group (5) CUMS + OX1R TM 5 treat group. For the operation of the lateral ventricle cannulation, the rats were anesthetized with chloral hydrate (0.3 g/kg, intraperitoneal), and the surgical operation was performed according to the previous description in our laboratory (1). The cannula was placed in the right ventricle of each rat (Riverward / China; stereotactic localization: 0.8 mm posterior and posterior, 2.0 mm lateral to the right, 3.8 mm deep). This was cement-fixed and recovered for five days after the casing was placed. The TM treatment was started on the sixth day of the cannula placement. Rats were randomly assigned to treatment groups. Test TM peptide were randomly assigned over multiple testing days. The TM treatment group was injected with the corresponding TM (100μg /8μl) per day and chronic unpredictable stimulation at the same time. The behavioural testing was performed after six consecutive days of administration. The test TM peptide (or saline) were blinded to the experimenter. All animals were given deep anesthesia, and then were killed, with their brains being collected for further biomarker analysis. Data collection and analysis in the experiments were performed in a blinded fashion.

Rat Brain Slice Preparation: The brains were isolated from decapitated animals and rapidly frozen on dry ice. Coronal brain sections (5 mm thickness for PLA technique and immunofluorescence staining experiments) were cut using a Jung CM 3000 cryostat microtome (Leica, Germany). The slices were thaw-mounted on gelatin-covered microscope slides, air-dried, and stored at −20 °C until use. PLA experiments: CUMS rats with TM treat and controls were blinded to the experimenter.

**Cell lines and chemicals**

The HEK293 cells were obtained from American Type Culture Collection (ATCC). All restriction enzymes were procured from New England Bio Labs. 8-OH-DPAT and 5-HT1AR antibodies (goat) were purchased from Sigma-Aldrich. Orexin A and OX1R antibodies (rabbit) were obtained from Abcam. Lipofectamine 2000 and Opti-MEM I was procured from Invitrogen Life Technologies. Coelenterazine h was obtained from Promega. HEPES-buffered phenol red-free medium and Dulbecco’s modified eagle medium were purchased from Gibco. Anti-HA-agarose was obtained from Pierce Chemical Co. Polyclonal horseradish peroxidase-conjugated goat anti-rabbit immunoglobulins/HRP was obtained from Zhong Shan Gold Bridge Biology Corporation (China). Anti-Myc and anti-HA antibodies were purchased from Cell Signalling Technology. SRE-luc, CRE-luc, and NFAT-RE-luc were obtained from Promega (Madison, WI, USA).

**Plasmid construction**

Plasmids pcDNA3.1 5-HT1AR, pcDNA3.1-OX1R and 3XHA-OX1R were obtained from the UMR cDNA Resource Center (University of Missouri-Rolla, USA). 5-HT1AR-ECFP, OX1R-ECFP, 5-HT1AR-EYFP, and OX1R-EYFP, which encoded an ECFP-tag and EYFP-tag were constructed. Enhanced cyan fluorescent protein (ECFP) and EYFP were attached to the C-termini of 5-HT1AR/OX1R by inserting the ORFs of 5-HT1AR/OX1R into the ECFP-N1 and EYFP-N1 vectors, respectively, resulting in 5-HR1AR-ECFP, OX1R-ECFP, 5-HT1AR-EYFP, and OX1R-EYFP. Myc-5-HT1AR was constructed as described previously (2). 5-HT1AR-Rluc and OX1R-Rluc were constructed as described; all of these constructs encode a C-terminal Renilla luciferase tag. A series of 5-HT1AR or OX1R mutants were generated by overlapping extension PCR using high fidelity Pfu polymerase and mutagenic primers. The mutagenic 5-HT1AR or OX1R cDNA was cut sequentially with Xho I and Bam HI or Xho I and EcoR I, and then ligated back into the pEGFP-N1. All reconstructed plasmids were verified by commercial DNA sequencing.

**Cell culture and cDNA transfection**

The HEK293 cells were obtained from American Type Culture Collection (ATCC). The cells were cultured in Dulbecco’s modified Eagle’s medium (DMEM) supplemented with 10% (v/v) fetal bovine serum (FBS; Invitrogen, Life Technologies) at 37 °C, 5% CO2. Transient transfections were performed with Lipofectamine 2000, according to the manufacturer’s protocol. HEK293 cells were transfected with pcDNA3.1-5-HT1AR, pcDNA3.1-OX1R, pcDNA3.1-5-HT1AR, and pcDNA3.1-OX1R to obtain the next generation of the cells by stably expressing 5-HT1AR, OX1R, 5-HT1AR, and OX1R. The screening was performed in 24-well plates with G418 (0.5 mg/mL) for 10 weeks. Receptor expression was assessed by performing Western blotting.

**Enzyme-linked immunosorbent assay (ELISA)**

One day before transfection, HEK293 cells were placed in 96-well plates at a density of 1 × 10^4^ cells per well. Transient transfection was performed for expressing Myc-5-HT1AR and/or HA-OX1R using Lipofectamine 2000. After 24 h, cells were fixed using 4% paraformaldehyde in phosphate-buffered saline (PBS) for 30 min at room temperature. The cells were washed three times with PBS, and nonspecific binding sites were blocked with the blocking buffer (3% dry milk). Then, cells were incubated with rabbit polyclonal anti-HA or anti-Myc primary antibodies overnight at 4 °C, subsequently washed with PBS three times, and further incubated with a peroxidase-conjugated goat/rabbit secondary antibody in DMEM for 1 h at 37 o C. Primary antibodies were used in 1:500 dilutions and secondary antibodies as 1:2000 dilutions in blocking buffer. The final substrate (200µl) 3,3’,5,5’-tetramethylbenzidine (Sigma-Aldrich) was added and incubated for 30 min at 37 o C, and the enzymatic reaction was stopped by adding 50 μL of 2% NH2SO4 solution. The orange color was produced when the substrate reacted with the peroxidase enzyme conjugated to the secondary antibody. The colorimetric optical density of each well was measured using an iMark Microplate reader (Bio-Rad, USA) at 450 nm. The optical density was maintained between 0.1 and 1.0 to avoid saturation.

**Intracellular cAMP level measurements**

HEK293-5-HT1AR, HEK293-OX1R, HEK293 5-HT1AR, and HEK293-OX1R stable cell lines were cultured in 24-well cell culture plates (1–2 ×10^6^). Intracellular cAMP levels were measured with a cAMP ELISA kit (Cell Biolabs, Inc., United States), according to the manufacturer's instructions. Intracellular cAMP was also assayed using an Epac cAMP BRET biosensor (3). Briefly, YFP-Epac-RLuc plasmids and receptors of interest were co-transfected into HEK293 cells. The cells were collected and distributed in a 96-well microplate after 24 h and cultured in HEPES-buffered phenol red-free medium for another 24 h. Cells were washed with PBS and resuspended in Dulbecco’s phosphate buffered saline (D-PBS). BRET was measured at room temperature. Cells were stimulated with 5-HT1AR and /or OX1R agonists (8-OH-DPAT 100 nM and/or orexin-A, 100 nM) for 5 min. BRET readings were collected by a Tristar LB941 plate reader (Berthold Technologies GmbH & Co., Germany).

**Western blotting**

Take 1.5 mm tissue samples of prefrontal cortex and hippocampus from normal, CUMS and TM treated CUMS rats. Transfer the tissue to a homogenizer and add RIPA buffer with protease inhibitor.

Bicinchoninic acid (BCA) assay was used to determine the protein concentration in supernatants, with BSA (1 mg/mL) as a standard. Then, the samples (10–20 μg of protein/lane) were separated by 10% SDS-PAGE followed by transfer to PVDF membranes. The proteins of interest were probed with primary antibodies against pCREB, CREB, BDNF, and β-catin. Primary antibodies were diluted 1:1000 with 5% BSA and secondary antibodies were diluted 1:5000. Enhanced chemiluminescence (ECL) kits were used to visualize and analyze protein bands. Films were scanned and bands were analyzed using a ChemiDoc MP Imaging System (Bio-Rad). Changes in CREB phosphorylation were calculated as the pCREB/total-CREB ratio, as previously described (6, 7).

**Proximity ligation assay (PLA)**

The interactions of 5-HT1AR/OX1R were detected in the native tissue using the Duolink II in situ PLA detection Kit (Sigma-Aldrich, USA) following the supplier’s instructions. Rat brain (hippocampus) sections of 5 μM thickness were used in the PLA method. A mixture of the primary antibodies [goat polyclonal anti-5-HT1AR antibody (1:100; Sigma-Aldric, USA) and rabbit polyclonal anti-OX1R antibody (1:100; Abcam)] was used for detecting 5-HT1AR/OX1R heterodimers together with PLA probes for detecting goat or rabbit antibodies. Punctate fluorescent signals were indicative of close proximity (∼10 nm) between 5-HT1AR/OX1R, which confirmed the formation of heterodimers by laser scanning confocal microscopy with an apochromatic 63X oil-immersion objective and 405-nm and 561-nm laser lines. Similar methods were used for identifying the presence of 5-HT1AR/OX1R heterodimers in HEK293 cells transfected with 5-HT1AR/OX1R expressing vectors. HEK293 cells expressing OX1R alone were used as negative controls.

For elucidating the effects of TM peptides on 5-HT1AR/OX1R dimerization, HEK293 cells were transfected with the plasmids, pcDNA3.1-5-HT1AR and pcDNA3.1-OX1R in 12-well plates. After 24 h, the cells were distributed into 6-well plates and treated for 6 h with HIV TAT–fused TM peptides (10 μM) corresponding to TM4 or TM5 of 5-HT1AR, or TM1 or TM5 of OX1R and then assayed for dimerization as described above.

**Bioluminescence resonance energy transfer (BRET) assay**

For monitoring constitutive 5-HT1AR/OX1R interactions, HEK293 cells were transfected with 5-HT1AR-Rluc and OX1R-EGFP or OX1R-Rluc and 5-HT1AR-EGFP plasmids at the ratios of 1:1, 1:2, 1:3, 1:4, 1:5, and 1:6 for expressing a constant amount of donor-labeled protein with increasing amounts of acceptor-labeled protein. After 24 h of post-transfection, cells were trypsinized and placed on a 96-well microplate for 24 h in HEPES-buffered phenol red-free medium (Invitrogen, Life Technologies). Coelenterazine h (5 μM; Promega) was added for BRET measurements using a Tristar LB941 plate reader (Berthold) with Rluc (400–475 nm) and EGFP (500–550 nm) filters. BRET signals were measured for monitoring the induced interactions of 5-HT1AR/OX1R, as described above, with slight modifications. Cells were transiently transfected with 5-HT1AR-Rluc and OX1R-EGFP and stimulated with agonists (100 nM 8-OH-DPAT or 100 nM Orexin A). For determining the effects of interfering peptides on 5-HT1AR/OX1R dimers, HEK293 cells were co-transfected with 5-HT1AR-Rluc and OX1R-EGFP (1:3), incubated with interference peptides corresponding to 5-HT1AR (TM4, TM4 R151A mutant, TM5 and TM6), or OX1R (TM1, TM4, TM5 and TM6), stimulated with or without agonists at 37 °C (10 μM) for 2h, and detected by BRET assay as described above.

**Fluorescence resonance energy transfer (FRET)**

The donor plasmid 5-HT1AR-ECFP and receptor plasmid OX1R-EYFP were co-transfected into HEK293 cells as the FRET channel. In addition, the donor and acceptor channels were used for eliminating the crosstalk into the FRET channel. FRET sample preparations must, therefore, include references of the donor in the absence of the acceptor (donor-only control) and acceptor in the absence of the donor (acceptor-only control). 5-HT1AR-ECFP and OX1R-EYFP were transfected into HEK293 cells as donor and acceptor channels, respectively for obtaining calibration coefficients and eliminate excitation and emission crosstalk. After 12–24 h, FRET signals were detected with a FRET Kit using a Leica AM TIRF MC system (Leica Microsystems). Calculation of FRET efficiency: EA is the apparent FRET efficiency. A, B, C correspond to the intensities of the three signals (donor, FRET, acceptor), and α, β, γ, and δ are the calibration factors generated by the acceptor only and donor-only references:

$$\text{EA}(i)=\frac{\text{B}-\text{A}\times\beta-\text{C}\times(\gamma-\alpha\times\beta)}{\text{C}\times(1-\beta\times\delta)}$$

**Design and synthesis of TM peptides**

Mutant TMs and peptides, derived from human 5-HT1R WT, mutant TMs, and OX1R WT were custom-synthesized, and their primary sequences are shown in Table 1. The identities of the TM peptide sequences were analyzed with liquid chromatography-mass spectrometry (LC-MS). HIV TAT (YGRKKRRQRRR) was fused at the N-terminus of even-numbered TMs and C-terminus of odd-numbered TMs to obtain the correct orientation for the inserted peptide because HIV TAT binds to phosphatidylinositol-(4,5)-bisphosphate on the inner surface of the membrane.

After custom synthesis, the identities of the TM peptide sequences were analyzed using an LC-MS system (Shimadzu2020 and Water1010). The molecular weights of 5-HT1A TM 4, 5, 6 and 4 mutants (R151A) were 4372, 4253, 4138 and 4171 Da, respectively. The molecular weights of OX1R TM 1, 4, 5, and 6 were 3788, 3765, 4209, and 4134.23Da, respectively.

Cells were incubated with the abovementioned peptides at 37°C for 2 h prior to performing BRET analysis, in situ PLA assays

**Mass spectrometry**

Mass spectrometry was performed for identifying 5-HT1AR/OX1R dimer interfaces in samples treated with TM peptides. Cells were transfected with 5-HT1AR or OX1R, and 48 h later, treated with or without the indicated HIV TAT-TM fused peptides (4 μM) for 60 min at 37 °C. Extracted proteins were immunoprecipitated using anti-5-HT1AR or OX1R antibodies. Protein A/G PLUS-agarose beads were incubated with proteins for 2 h and washed four times with lysis buffer. The 5-HT1AR or OX1R complexes were eluted from the beads. The 5-HT1AR or OX1R complex was analyzed using an AXIMA matrix-assisted laser desorption/ionization time-of-flight mass spectrometer, (MALDI-TOF MS) system (Shimadzu). We also investigated whether the dimeric interface changes during receptor activation. Similar methods were used for detecting the activities of 5-HT1AR/OX1R heterodimers after stimulating with 8-OH-DPAT or orexin A.

**NFAT-RE, CRE, SRE and SRF-RE Luciferase Reporter Assay**

The activities of NFAT-RE (nuclear factor of activated T-cells-response element), CRE (cAMP-response element), SRE (serum response element) and serum response factor response element (SRF-RE) were detected in HEK293-5-HT1AR, HEK293-OX1R, HEK293-5-HT1AR and HEK293-OX1R stable expression cells for studying the effects of 5-HT1AR/OX1R heterodimers on downstream signaling. NFAT-RE, CRE, SRE and SRF-RE luciferase reporter assays were performed by transfecting the 5-HT1AR, OX1R, or 5-HT1AR/OX1R cells with NFAT-Luc or CRE-Luc, or SRE-Luc or SRF-RE-luc, together with pRL-Tk. The cells were starved and stimulated with 8-OH-DPAT (100 nM) or/and orexin-A (100 nM) for 6 h prior to harvest at 24 h after transfection. These experiments were performed as described previously (5).

**Computational models**

The 3-D structure of OX1R was retrieved from the PDB database (PDB ID 4ZJ8). The 3-D structure of 5-HT 1AR was constructed by homology modeling using the homologous GPCR and 5-HT 1B receptor (PDB ID 5V54) as templates. The homology modeling was performed using MODELLER 9.20. The structure models of 5-HT1AR/OX1R heterodimer were assembled in active and inactive states by manually docking the two GPCR models, according to the known information on the dimerization interface as illustrated in Supplementary Figure 5. For a clear picture, some intracellular and extracellular loops were removed.

Statistical analysis

All data are shown as means ± SEM. Data are presented and analyzed using GraphPad Prism 5.0 software. Sigmoidal curves were fitted to the dose-response data using nonlinear regression. Statistical analysis was performed using one-way analysis of variance followed by Tukey's multiple comparison post-test. Differences between the means were considered statistically significant at P < 0.05.

REFERENCES

1. Yan XG, Cheng BH, Wang X, Ding LC, Liu HQ, Chen J, et al. Lateral intracerebroventricular injection of Apelin-13 inhibits apoptosis after cerebral ischemia/reperfusion injury. Neural Regen Res. 2015 May;10(5):766-71.
2. Xue Q, Bai B, Ji B, Chen X, Wang C, Wang P, et al. (2018): Ghrelin Through GHSR1a and OX1R Heterodimers Reveals a Galphas-cAMP-cAMP Response Element Binding Protein Signaling Pathway in Vitro. Frontiers in molecular neuroscience. 11:245.
3. Ji B, Liu H, Zhang R, Jiang Y, Wang C, Li S, et al. (2017): Novel signaling of dynorphin at kappa-opioid receptor/bradykinin B2 receptor heterodimers. Cellular signalling. 31:66-78.
4. Cai X, Bai B, Zhang R, Wang C, Chen J (2017): Apelin receptor homodimer-oligomers revealed by single-molecule imaging and novel G protein-dependent signaling. Scientific reports. 7:40335.
5. Bai B, Chen X, Zhang R, Wang X, Jiang Y, Li D, et al. (2017): Dual-agonist occupancy of orexin receptor 1 and cholecystokinin A receptor heterodimers decreases G-protein-dependent signaling and migration in the human colon cancer cell line HT-29. Biochimica et biophysica acta Molecular cell research. 1864:1153-1164.
6. Liu H, Tian Y, Ji B, Lu H, Xin Q, Jiang Y, Ding L, et al. Heterodimerization of the kappa opioid receptor and neurotensin receptor 1 contributes to a novel β-arrestin-2-biased pathway. Biochim Biophys Acta. 2016 Nov;1863(11):2719-2738. d
7. Chen J, Chen X, Li S, Jiang Y, Mao H, Zhang R, Ji B, Yan M, Cai X, Wang C. Individual phosphorylation sites at the C-terminus of the apelin receptor play different roles in signal transduction. Redox Biol. 2020 Sep;36:101629. doi: 10.1016/j.redox.2020.101629.

**
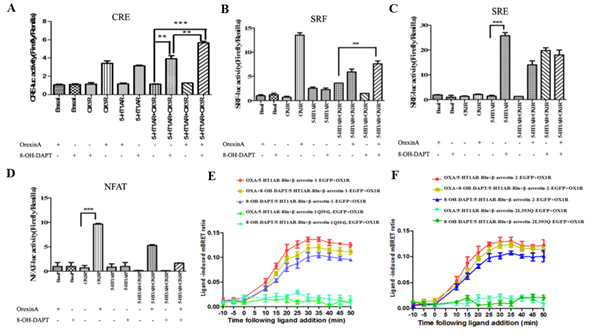
**

**Supplementary Figure 1.**

**Effects of 5-HT1AR/OX1R heterodimerization on NFAT, CRE, SRF and SRE activities and β-arrestin recruitment.**

Twenty-four hours after transfection with CRE-luc (A), SRF-Luc (B), SRE-luc (C) NFAT-luc (D), with pRL-TK, HEK293-5-HT1AR, HEK293-OX1R and HEK293-5-HT1AR/OX1R cells were treated with 8-OH-DPAT (100 nM) and/or orexin-A (100 nM) as indicated for 6 h prior to harvest. Firefly and Renilla luciferase activity were assayed using a Dual-Luciferase Reporter Assay System according to the manufacturer's instructions. The ratio of firefly luciferase luminescence to that of Renilla luciferase was determined. Results were expressed as the mean ± SEM of four independent experiments performed in duplicate. Statistical analysis was performed by one-way ANOVA followed by Tukey's multiple comparison post hoc test. ** p < 0.01, *** p < 0.001, HEK293 cells expressing 5-HT-1AR and OX1R by no agonist treated vs. cells co-expressing 5-HT1AR/OX1R by 8-OH-DPAT and orexin-A treated.

HEK293 cells transiently co-expressing 5-HT1AR-Rluc and β-arrestin1-EGFP/ β-arrestin1Q394L-EGFP (E) or β-arrestin2-EGFP/ β-arrestin2 L393Q-EGFP (F) with of OX1R were monitored by extended BRET for 10 min at 37 °C to generate kinetic curves. Following the addition of 8-OH-DPAT (100 nM) and/or orexin-A (100 nM), monitoring was continued for an additional 50 min. Data represent means ± SEM of four independent experiments (n=4).


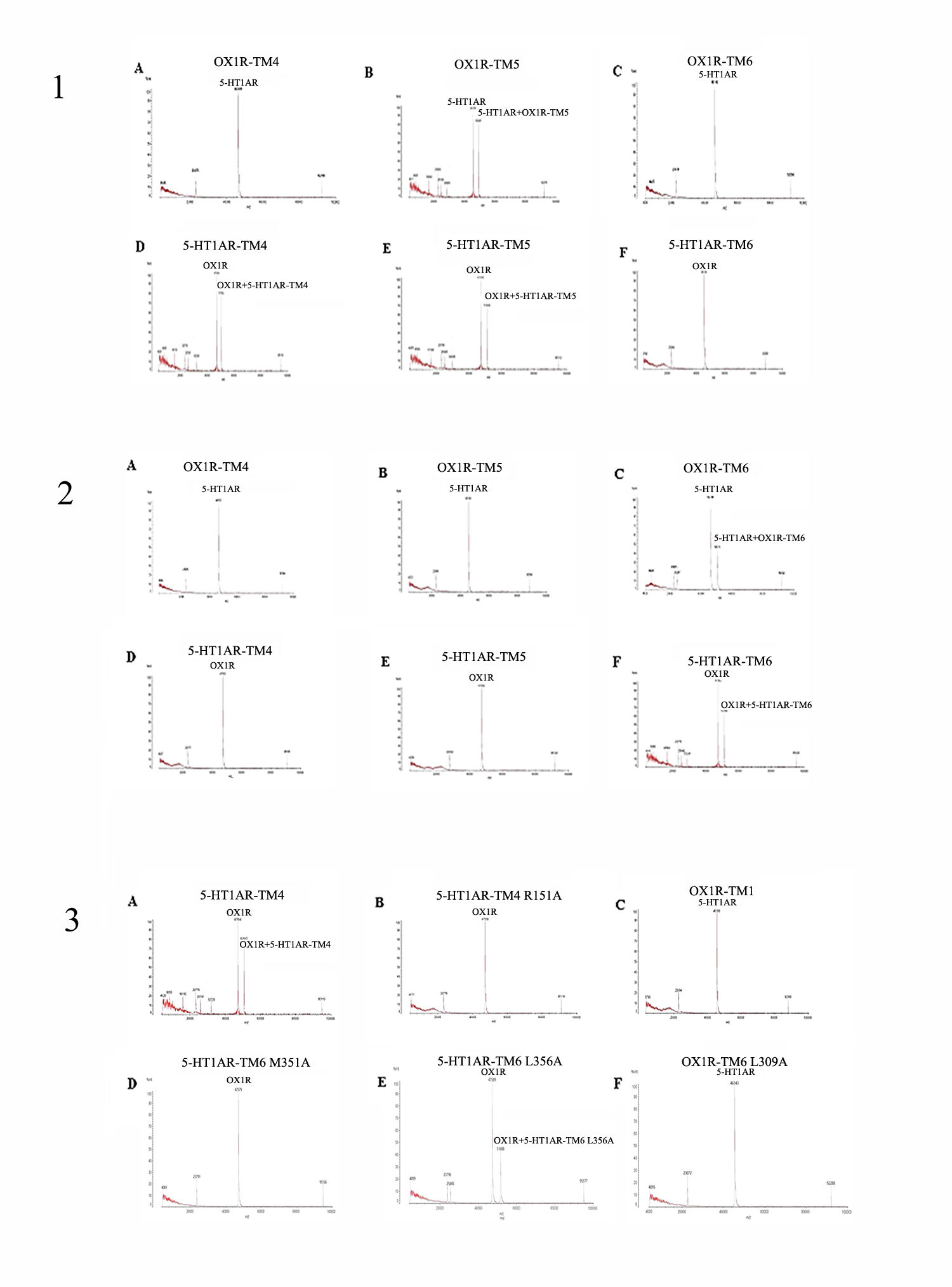


**Supplementary Figure 2.**

**Identification of the interaction interface of 5-HT1AR/OX1R heterodimerization using TM peptides and mass spectrometry.**

When the density of HEK293 cells was grown to 70%-80%, pcDNA3.1 (+)-OX1R or pcDNA3.1 (+)-5-HT1AR was transfected into cells by cell transfection. After 48 h, the designated HIV TAT-TM fusion peptide (4 μM) was incubated at 37 ° C for 60 minutes. Stimulate with or without agonist (100 nM） for 15 minutes depending on the actual situation.

In the mass spectrum, the abscissa (X) represents the mass charge ratio (M/Z) of ions, and the mass charge ratio increases from left to right. The value represented by the abscissa is the mass of the ion; The ordinate (Y) represents the intensity of the ion current.

1. Analysis of the interface between 5-HT1AR/OX1R heterodimers (without ligands) using interface peptides and mass spectrometry

A, OX1R-TM4: TM4, 5-HT1AR and 5-HT1AR dimer were detected (TM4 MW = 3765 Da) B, OX1R-TM5: TM5, 5-HT1R, [5-HT1AR+TM5] and 5-HT1AR dimer were detected (TM5 MW= 4209 Da) C, OX1R-TM6: TM6, 5-HT1AR and 5-HT1AR dimer were detected (TM6 NM=4134 Da) D, 5-HT1AR –TM4: TM4, OX1R, [OX1R+TM4], OX1R dimer were detected (TDM4 NW= 4025, E, 5-HT1AR –TM5: TM5, OX1R, [OX1R+TM5], OX1R dimer were detected (TM NW=4253) F, 5-HT1AR –TM6: TM6, OX1R, OX1R dimer were detected (TM NW=4138). 5HT1AR MW=46.176 kDa，OX1R MW=47.536 kDa.

2. Analysis of the interface between 5-HT1AR/OX1R heterodimers (with ligands) using interface peptides and mass spectrometry

A,OX1R-TM4: TM4, 5-HT1AR and 5-HT1AR dimer were detected B, OX1R-TM5: TM5, 5-HT1R and 5-HT1AR dimer were detected C, OX1R-TM6: TM6, 5-HT1AR [5-HT1AR+TM6] and 5-HT1AR dimer were detected D, 5-HT1AR–TM4: TM4, OX1R, OX1R dimer were detected, E, 5-HT1AR–TM5: TM5, OX1R, OX1R dimer were detected F, 5-HT1AR –TM6: TM6, OX1R, [OX1R+TM6] and OX1R dimer.

3. Analysis of the interface between 5-HT1AR/OX1R heterodimers using mutant interface peptides and mass spectrometry.

A, 5-HT1AR-TM4: TM4, OX1R, [OX1R+TM4] and OX1R dimer were detected B, 5-HT1AR-TM4 R151A: TM4 R151A, OX1R and OX1R dimer were detected C, OX1R-TM1: TM1, 5-HT1R, and 5-HT1AR dimer were detected D, 5-HT1AR-TM6 M351A: TM6 M351A, OX1R, OX1R dimer were detected E, 5-HT1AR-TM6 L356A: TM6 L356A, OX1R, [OX1R+M6 L356A], OX1R dimer were detected F, OX1R-TM6 L309A: TM6 L309A, 5-HT1AR, and 5-HT1AR dimer were detected.


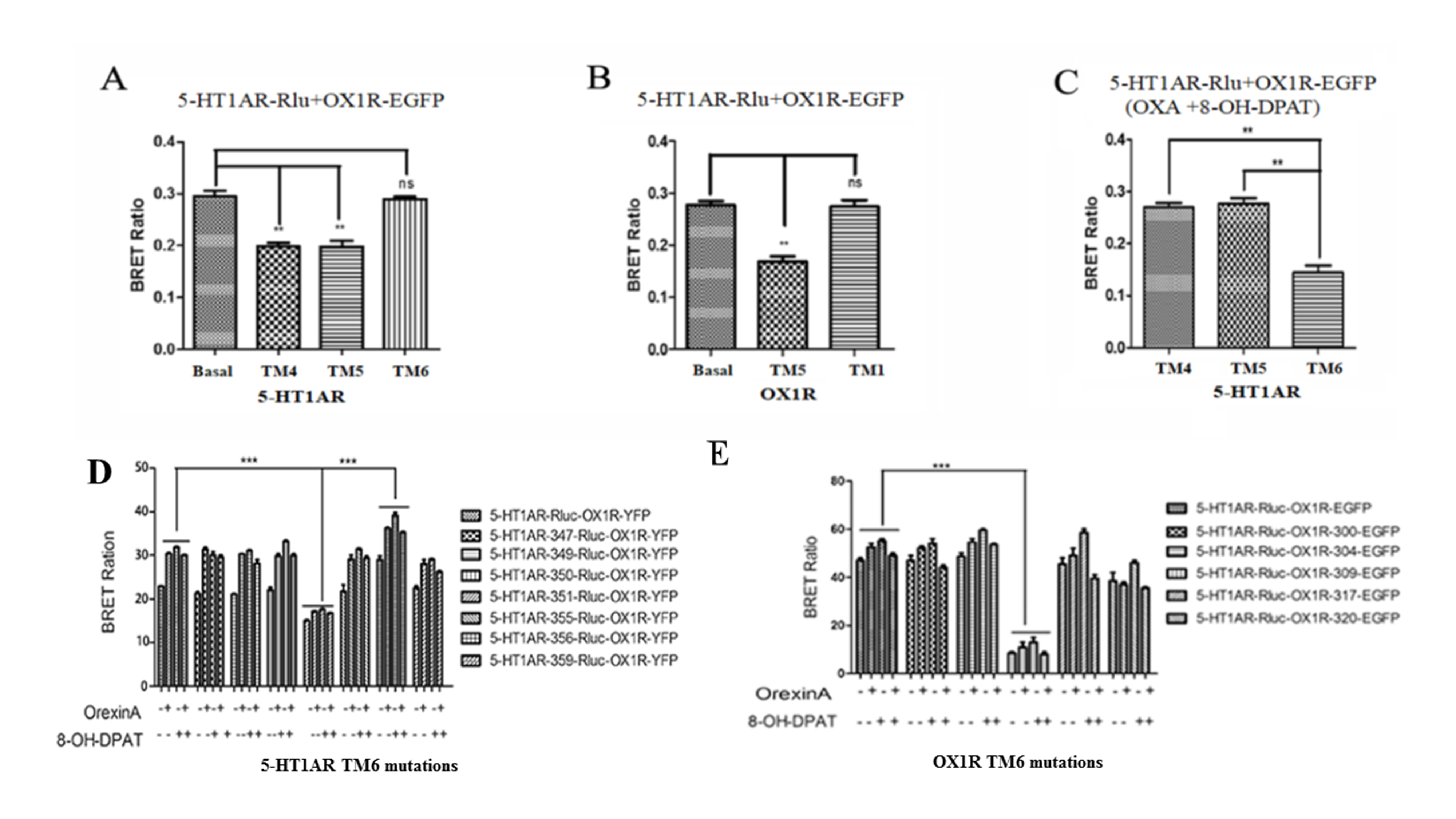


**Supplementary Figure 3.**

**The effects of TM peptide on 5-HT1AR / OX1R heterodimer and 5-HT1AR-TM4 R151A and OX1R-TM4 M176A mutants were evaluated by BRET experiments.**

HEK293 cells were co-transfected with 5-HT1AR-Rluc and –OX1R EGFP (1:3) and incubated at 37 °C for 2 h with HIV TAT–fused TM peptides (10 μM) corresponding to respectively TM4, or TM5 or TM6 of 5-HT1AR (A) and also TM1 or TM5 of OX1R (B). After incubated with interference peptides corresponding to TM4, TM5, TM6 of 5-HT1AR at 1h, then stimulated with 100 nM 8-OH-DPAT and 100 nM orexin-A (C). BRET ratios were analyzed and expressed as means ± SEM of four experiments (n=4) (one-way analysis of variance; ns: not significant, **, p < 0.01 vs. control group).

Detection of a series of 5-HT1AR-TM6 and OX1R-TM6 mutants by BRET. The agonist stimulation of 5-HT1AR L347A 6, I349A, I350A, M351A, W355A, L356A and L359A mutants in TM6 reduced or increased 5-HT1AR/OX1R dimers’ BRET signals (Figure. 3S, D). Experimental Evaluation of OX1R-TM6 Interface six Mutation (Figure. 3S, E) Using BRET, four independent experiments were performed with triplicate samples and the results were expressed as the mean ± SEM of four experiments (n=4) (* p < 0.05 ** p < 0.01, *** p < 0.001, NS, not significant versus WT 5-HT1AR/OX1R dimer group). Figure 3F, Interfacial transition model diagram of 5-HT1AR/OX1R heterodimer from TM4 and TM5 without ligands (inactive state) to mainly TM6 with ligands in the active conformation.


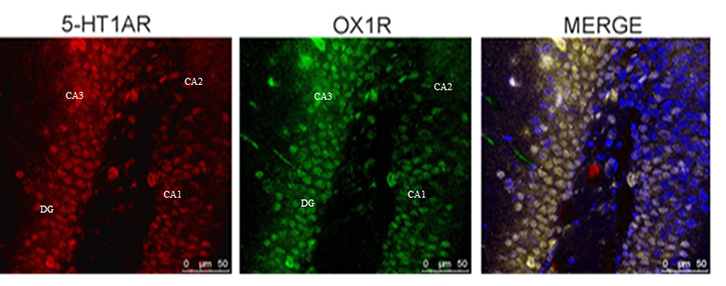


**Supplementary Figure 4.**

Double-immunofluorescence staining and confocal images to detect co-localization of 5-HT1AR/OX1R in the neuronal cells of the whole hippocampus are displayed in Fig, S4.

Red is 5-HT1AR; green is OX1R; blue is nucleus; yellow is co expression of 5-HT1AR and OX1R. Scale bars: 50 μm.


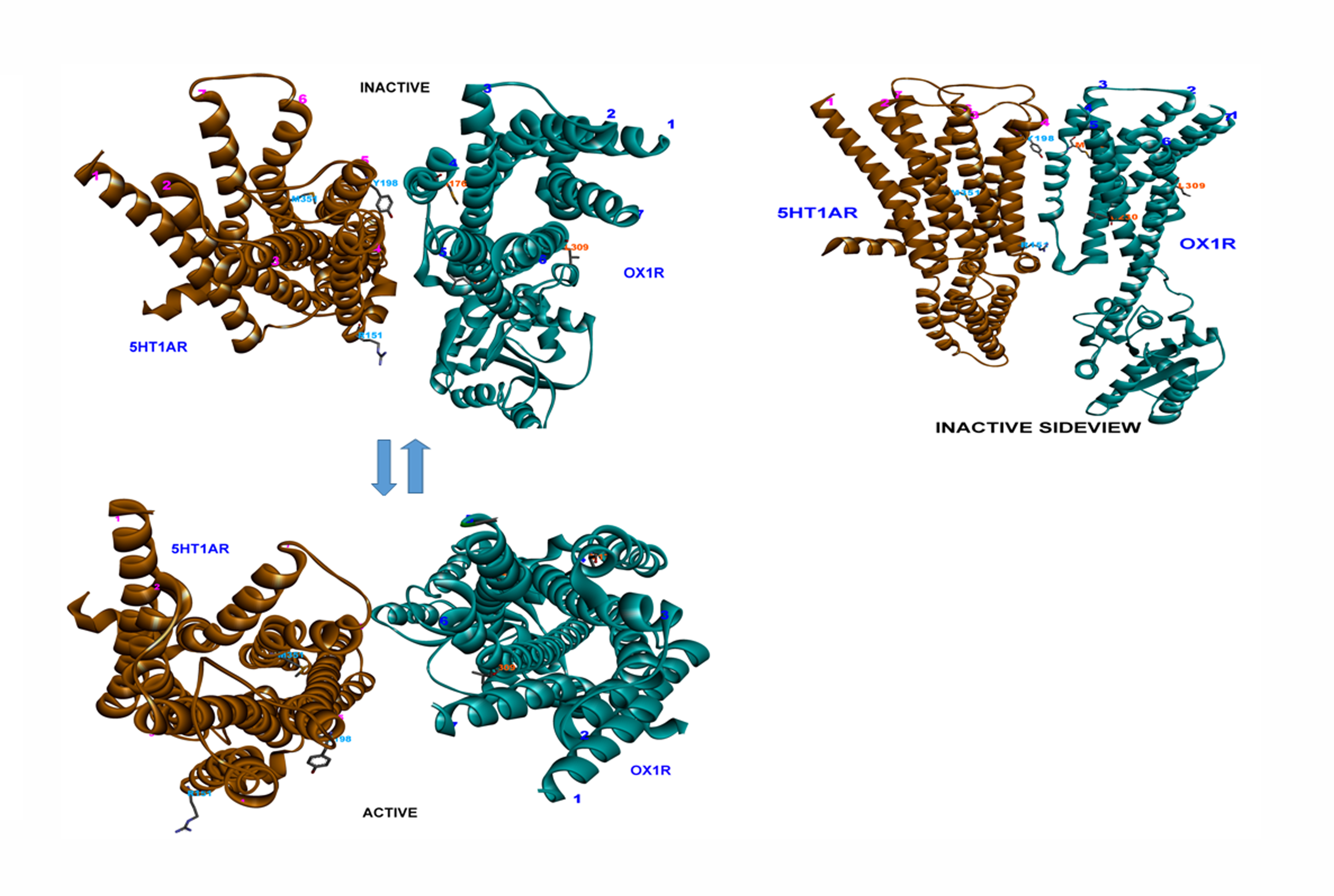


**Supplementary Figure 5**

**Structure models of 5-HT1AR/OX1R heterodimer**

The structure models of 5-HT1AR/OX1R heterodimer were assembled in active and inactive states by manually docking the two GPCR models, according to the known information on the dimerization interface as illustrated in Supplementary Figure 5. For a clear picture, some intracellular and extracellular loops were removed.
